# Reductive Stress-Induced Mitochondrial Dysfunction and Cardiomyopathy

**DOI:** 10.1155/2020/5136957

**Published:** 2020-05-29

**Authors:** Wei-Xing Ma, Chun-Yan Li, Ran Tao, Xin-Ping Wang, Liang-Jun Yan

**Affiliations:** ^1^Department of Pharmaceutical Sciences, UNT System College of Pharmacy, University of North Texas Health Science Center (UNTHSC), Fort Worth, Texas 76107, USA; ^2^Qingdao University of Science and Technology, 266042 Qingdao, Shandong, China; ^3^Shantou University Medical College, 515041 Shantou, Guangdong, China; ^4^Qingdao Municipal Center for Disease Control & Prevention, 266034 Qingdao, Shandong, China

## Abstract

The goal of this review was to summarize reported studies focusing on cellular reductive stress-induced mitochondrial dysfunction, cardiomyopathy, dithiothreitol- (DTT-) induced reductive stress, and reductive stress-related free radical reactions published in the past five years. Reductive stress is considered to be a double-edged sword in terms of antioxidation and disease induction. As many underlying mechanisms are still unclear, further investigations are obviously warranted. Nonetheless, reductive stress is thought to be caused by elevated levels of cellular reducing power such as NADH, glutathione, and NADPH; and this area of research has attracted increasing attention lately. Albeit, we think there is a need to conduct further studies in identifying more indicators of the risk assessment and prevention of developing heart damage as well as exploring more targets for cardiomyopathy treatment. Hence, it is expected that further investigation of underlying mechanisms of reductive stress-induced mitochondrial dysfunction will provide novel insights into therapeutic approaches for ameliorating reductive stress-induced cardiomyopathy.

## 1. Introduction

Redox imbalance (RI), as a hallmark event in cardiac and other pathophysiology [1], results from the perturbance of balance between oxidants and antioxidants [2], which can lead to either reductive stress or oxidative stress [3]. The redox state of cells is established by four redox pairs including NAD^+^/NADH, NADP^+^/NADPH, reduced glutathione (GSH)/oxidized glutathione (GSSG), and FAD/FADH_2_ [4, 5]. In comparison with oxidative stress, reductive stress has recently gained more interest, and many related investigations have been published [6–9] since the concept was first introduced [10]. Reductive stress is considered to be a double-edged sword in terms of antioxidation and disease induction. As many relative mechanisms are still unclear, the area of reductive stress is certainly worth of further investigations. Remarkably, the striking discovery that reductive stress can cause cardiomyopathy by protein aggregation published in Cell in 2007 [11] has been a driving force for this area of research.

A key difference between reductive stress-induced and oxidative stress-induced cardiomyopathy is the enzymes and pathways controlled or regulated by certain factors. For instance, inhibition of NADPH oxidase activities by NecroX-7 can prevent oxidative stress-induced cardiomyopathy [12]. Sulforaphane prevents the deterioration of cardiomyopathy by reversing oxidative stress-induced inhibition of LKB/AMPK pathway [13]. Fibroblast growth factor 19 prevents the heart against oxidative stress-induced cardiomyopathy by activating the AMPK/nuclear factor erythroid 2-related factor 2 (Nrf2)/HO-1 pathway [14].

By comparing with oxidative stress-induced cardiomyopathy, in this review, we seek to summarize the studies focusing on reductive stress-induced mitochondrial dysfunction and cardiomyopathy published in the past five years. In the section of cardiomyopathy, reductive stress-inducing factors including heat shock protein 27 (Hsp27 or HspB1), alpha-B crystalline (CryAB or HspB5), and Nrf2 were specifically elaborated. Moreover, current insights into dithiothreitol- (DTT-) induced reductive stress, and the side effects of DTT were also discussed. In addition, under reductive stress conditions, activities of NADPH-dependent reductases such as glutathione reductase (GR) and thioreductase-2 (TrxR2) are closely related to reactive oxygen species (ROS) reactions [15], which have been established to contribute to mitochondrial dysfunction. Therefore, relevant studies and discussions are presented as well. It should be noted that our review is not meant to exhaust all the possible mechanisms or signaling pathways of reductive stress documented in the literature.

## 2. Redox Imbalance (RI) and Mitochondrial Dysfunction

**Figure 1:**
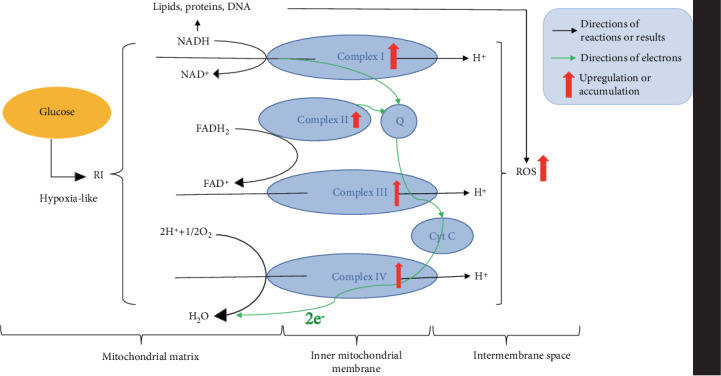
Interrelationship between RI and mitochondrial dysfunction. NAD^+^/NADH imbalance is the main cause leading to lipids, proteins, and DNA damage and triggering the increase in ROS production by the electron transport chain [16–19]. ROS are metabolic by-products generated by mitochondria that can damage macromolecules by structurally altering protein amino acids leading to formation of protein carbonyls, DNA mutation (e.g., single- and double-strand breaks, inter/intrastrand cross-links, and DNA-protein cross-links), and lipid peroxidation [29, 30].

Many studies currently focus on the interrelationship between RI and mitochondrial dysfunction. They are summarized and graphically presented in Figure 1. RI increases mitochondrial ROS production by upregulating the activities of complexes I to IV [16] and impacts the NAD^+^/NADH balance leading to damage to lipids, proteins [17, 18], and DNA [19]. Impaired oxidation of NADH to NAD^+^ by the electron transport chain (ETC) is an adaptive mechanism of hypoxia, analogous to the “hypoxia-like” RI resulting from increased flux of glucose [20, 21]. “Hypoxia-like” RI, often induced in vitro by cobalt [22], is the cytosolic metabolic imbalance due to reductive stress and increased superoxide and nitric oxide production [23]. Both hypoxia and “hypoxia-like” RI result in a loss of essential sterols and unsaturated fatty acids, but the basis for these alterations are disparate [22]. The function of ETC can be impacted by the imbalance between the generation of ROS and oxidation of ETC components, which can alter the membrane permeability, increase the heteroplasmic mitochondrial DNA, and finally weaken the mitochondrial defense system [24]. Moreover, the spontaneous DNA damage caused by mitochondria-derived ROS is able to activate the cycle of escalating ROS production, oxidative damage, senescent cell accumulation, and age-related pathology [25]. The extent of DNA damage paralleled the oxidation of cellular GSH and induction of oxidative stress [26]. Therefore, energy failure and RI can also result from mitochondrial depletion of DNA [27]. In response to DNA damage, the cell activates complicated and conserved kinase-involved signaling response termed DNA damage response to protect genomic stability; and once DNA damage is beyond repair, the cell initiates the apoptotic mechanism resulting in the demise of the damaged cell [28].

## 3. Reductive Stress-Induced Mitochondrial Dysfunction

**Figure 2:**
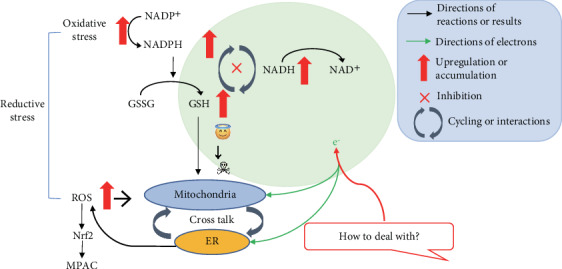
Interrelationship between reductive stress and mitochondrial dysfunction. Under reductive stress, abnormally increased electron pressure caused by increases of NADH, NADPH, and GSH leads to mitochondrial dysfunction [38]. Oxidative protein folding in the ER leads to the release of ROS via mitochondria-ER cross talk [44], which activates Nrf2 [46]. Nrf2-antioxidant response element signaling enhanced reductive stress in the human mutant protein aggregation cardiomyopathy (MPAC) [61]. So, how to deal with the excess high-energy electron is a critical step for the transition from cell damage to cell protection.

Reductive stress, first introduced in 1987 [10], is described as an excess of reducing equivalents, in the forms of NAD(P)H and/or glutathione, in the presence of intact oxidoreductive systems [31, 32]. NADPH-producing reactions are trigged under oxidative stress [33], because isocitrate dehydrogenase reaction with the release of NADH in the TCA cycle is essential for the generation of reducing power, which defends against oxidative stress [34]. NADPH is the driving energy source for removing peroxide by glutathione- and thioredoxin-dependent antioxidant system [35]. Increased NADPH accelerates the reduction of GSSG to GSH, and the elevated level of GSH can abundantly provide thiol group to the detoxification reactions [36], which also serves as an important antioxidation mechanism [37]. In Figure 2, reductive stress is presented as an aberrantly increased electron pressure, and it can occur as a result of pathological processes leading to an excess of electrons with high-energy compounds, and a failure of mechanisms for handling this rise in electron pressure, or a combination of both [38]. The rate of the mitochondrial ROS production is connected with the level of reduction of electron carriers capable of transferring electrons to O_2_ [39]. Mitochondrial ROS are generated when electrons leak from the ETC resulting in univalent reduction of O_2_ to superoxide, which contributes to the production of additional ROS such as hydrogen peroxide (H_2_O_2_) and hydroxyl radical (OH·) [40]. Reductive stress also can result in ROS production, by controlling mitochondria to utilize the abundance of reducing equivalents or by perturbing protein folding and endoplasmic reticulum (ER) function [41–43]. ER, containing diverse systems to constrain ROS accumulation [44], is much more oxidizing than other cellular compartments and is more vulnerable to reductive stress [45]. In fact, there is a redox cross talk between mitochondria and ER [44]. Oxidative protein folding in the ER leads to the release of ROS as by-products, which can be utilized to activate some transcriptional factors such as nuclear erythroid 2-related factor 2 (Nrf2) [46]. Electrons from aerobic breakdown of glucose are mainly stored in NADH for oxygen reduction and ATP generation. GSH and NADPH accumulation are closely connected with NADH metabolism [47, 48]. GSH upregulation is considered to be a protective mechanism, at least, when followed by an oxidative stimulus [49]. However, aberrant increase in GSH/GSSG ratio leads to reductive stress [37, 50] that could trigger mitochondrial dysfunction and cytotoxicity [51–53] and enhance maladaptive responses [41]. It should be pointed out that mitochondrial activity impacted by antioxidant-induced reductive stress is initially hampered by a low dose of antioxidants (0.003-0.013%), rather than by a high dose of antioxidants (0.03-0.1%) [54]. Low dose of antioxidants neutralizes ROS, inhibits the glycolysis, and finally decreases pyruvate for TCA cycle [55]. Therefore, reductive stress at the onset of pathology could evolve into oxidative stress later in disease progression [56]. However, one recent article demonstrates that, particularly in aged tissues, oxidative stress appears more prevalent than reductive stress, giving the impression that reductive stress is not a cause of mitochondrial oxidative stress in aging-related diseases [57]. Nonetheless, it should be noted that one should not focus just on oxidative stress, but also consider the pathways that are altered by reductive stress [58]. In healthy cells, ETC generates ATP and simultaneously recycles mitochondrial NADH to NAD^+^; while in the presence of a dysfunctional ETC, glycolysis can compensate the insufficiency of ATP [59]. However, NAD recycling should be the critical step for cell proliferation, because many pathways produce NADH as a metabolic factor [60].

## 4. Current Insights into the DTT-Induced Reductive Stress

**Figure 3:**
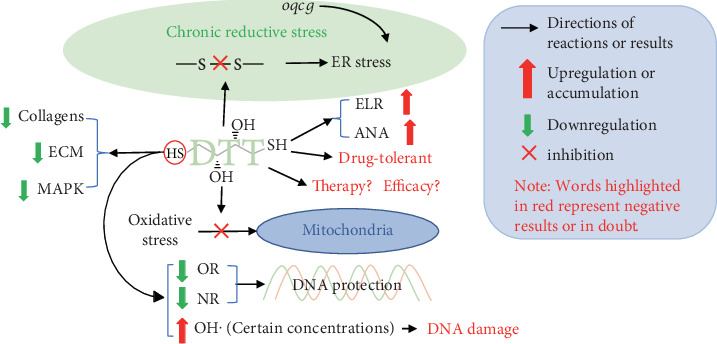
DTT-induced reductive stress and its potential risks. DTT induces chronic reductive stress and breaks disulfide bond [67, 68], which leads to ER stress [72]. Moreover, the concentration of DTT is closely related to DNA protection or DNA damage [74]. In addition, further investigations on its drug-tolerance and therapy as well as efficacy should also be highly warranted [63, 77–80].

DTT is a strong reducing agent, which can protect mitochondria from oxidative stress, radiation exposure, and mitochondrial damage [62, 63]. Mitochondrial dysfunction along with depletion of reduced glutathione can be recovered via DTT administration [64]. DTT also increases electrolyte leakage rate (ELR) and antioxidant enzymes activities (ANA) [65]. A previous study showed that, under DTT treatment, cytosolic redox active proteins become partially oxidized. However, under H_2_O_2_ treatment, ER-resident redox active proteins become oxidized with H_2_O_2_ and reduced after DTT treatment [66]. The main function of DTT is preventing disulfide bond formation [67, 68], disrupting protein folding in the ER, and being widely used as a chemical tool to promote reductive stress [3, 69–71]. Oxidative quality control genes (*oqcg*) modulate this ER stress in the presence of chronic reductive stress, not acute reductive stress [72]. Moreover, ER stress induced by DTT does not elevate the pool of secretory pathway. Rather, the reductive stress destabilizes a select set of proteins including collagens, the components of extracellular matrix (ECM), and mitogen-activated protein kinase (MAPK) signaling pathway targets [73]. In addition, although DTT is widely used, the side effect of DTT should be noted. Thiol is effective in protecting DNA against irradiation damage, which is thought to be due to its ability to scavenge ROS and reactive nitrogen species (RNS). However, at certain concentrations, thiols have the ability to produce oxidative species, such as OH·, leading to DNA breaks and other impairments in DNA molecules, which could further be connected with chromosome damage and cell apoptosis [74]. As DTT is often one of cell culture media components and can be added directly to the medium [75], studies have demonstrated that DTT lacks selectivity and spatial resolution [76] and leads to cellulose-anchored biofilm formation in Mycobacterium tuberculosis cultures, which contain metabolically active but drug-tolerant bacteria [77, 78]. Importantly, in certain studies, while DTT has been implemented as a therapy-oriented approach or treatment of some syndromes and complications [63, 79], its efficacy remains poorly understood [80]. Therefore, we think it is necessary to summarize the current studies to highlight the potential risks of DTT-involved approaches as presented graphically in Figure 3.

## 5. Reductive Stress and ROS

**Figure 4:**
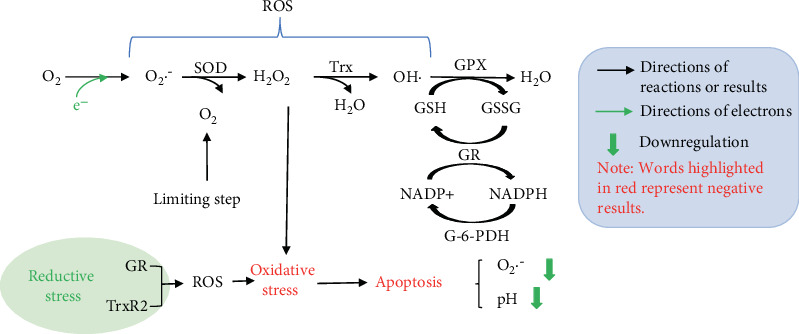
Potential mechanisms involved in reductive stress and ROS. Under reductive stress condition, the generation of ROS is attributed to NADPH-dependent reductases such as GR and TrxR2 when electron acceptors are impeded, which leads to apoptosis [15].

ROS, the products of partial O_2_ reduction, such as superoxide anion (O_2_·^−^), H_2_O_2_, and OH·, can induce necrotic death by producing oxidative stress. Exogenous or endogenous H_2_O_2_-induced apoptosis leads to a significant drop in the intracellular pH and O_2_·^−^ concentration [81, 82]. Moreover, during reductive stress, NADPH-dependent reductases such as GR and TrxR2 can directly generate ROS when the natural electron acceptors are hampered (Figure 4). The capacity of recombinant TrxR1 to generate NADPH-dependent H_2_O_2_ was 8-fold higher than recombinant GR. Lower GSH/GSSG levels in the matrix, whereby Trx is present at micromolar levels, could indicate that depletion of oxidized Trx might occur more readily than depletion of GSSG [15]. In contrast to GSH-related systems, removing H_2_O_2_ without oxidation of NAD(P)H is useless for the alleviation of the reductive stress [83]. Increased O_2_·^−^ and H_2_O_2_ levels can be viewed as creating oxidative stress or reductive stress depending upon the relative abundance of redox-coupled species [84]. O_2_·^−^ is dismutated by superoxide dismutase (SOD) resulting in H_2_O_2_ formation, and then H_2_O_2_ is detoxified by catalase or glutathione peroxidase (GPX) [85]. GPX, when knocked down, not only induces oxidative stress indicated by the increase of ROS but also causes reductive stress characterized by an elevation of GSH/GSSG [86].

## 6. Reductive Stress-Induced Cardiomyopathy

**Figure 5:**
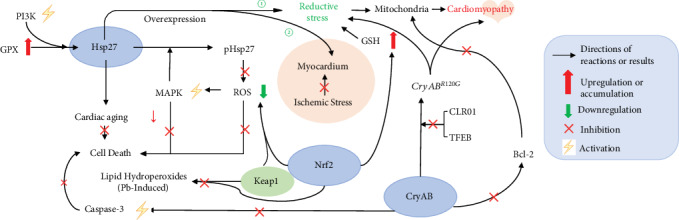
Signaling pathways potentially involved in reductive stress-induced cardiomyopathy. (1) Hsp27 constrains cardiac cell death, and phosphorylated Hsp27 (pHsp27) decreases ROS [94–96]. However, overexpression of Hsp27 leading to cardiomyopathy or protecting myocardium is controversial [95, 97–101]. (2) CryAB protects against caspase-3 activation and Bcl-2 [113] and maintains mitochondrial function [112]. *CryAB^R120G^* leads to cardiomyopathies via reductive stress [116, 117]. CLR01 and transcriptional factor EB (TFEB) protect against *CryAB^R120G^*-induced cardiomyopathy [119, 121]. (3) Nrf2 promotes GSH synthesis and decreases ROS [125]. However, continual activation of Nrf2 may contribute to chronic reductive stress and cardiomyopathy [61, 127].

In the heart, reductive stress has been connected with mitochondrial dysfunction, heart failure, ischemia-reperfusion injury, and cardiomyopathy, which all are the pathological conditions associated with oxidative stress [87]. Therefore, reductive stress is as important as oxidative stress in ischemic cardiac injury. It should be noted that mitochondrial morphological abnormalities cannot be identified in some studies in spite of the presence of severe cardiomyopathy and mitochondrial dysfunction [88]. Here we would like to focus on three cardiomyopathy-related factors: Hsp27, CryAB, and Nrf2 (Figure 5).

### 6.1. Hsp27-Induced Cardiomyopathy

Hsp27-induced cardiomyopathy could be attributed to the increase of GPX and mediated by activation of Class III phosphoinositide 3-kinase (PI3K) via a prolonged autophagy activation [89, 90]. Hsp27 also ameliorates cardiac aging, which involves antioxidation and mitophagy activation [91]. Moreover, phosphorylated Hsp27 (pHsp27) is catalyzed by one of the downstream targets of MAPK [92]. Activation of MAPK by ROS is also proven to protect cells against death [93]. Although pHsp27 decreases ROS accumulation and could constrain cardiac cell death [94–96], overexpression of Hsp27 can lead to reductive stress and contributes to cardiomyopathy (Figure 5) [97, 98]. However, other studies showed that overexpression of Hsp27 could protect myocardium during ischemic stress [95, 99–101]. Therefore, Hsp27, with its potent antiaggregation activity [102], may serve as an important indicator of the risk assessment and prevention of developing heart lesion as well as a target for cardiomyopathy treatment [103, 104]. Further studies are needed to clarify if increased Hsp27 is actually beneficial and is in response to stress exposure [105].

### 6.2. CryAB and CryAB^R120G^-Induced Cardiomyopathy

CryAB, an ER chaperone [106], is substantially expressed in the heart, where it constitutes as much as 5% of total heart protein [107]. CryAB, with its wide-spectrum chaperone activities [108], promotes the folding of multipath transmembrane proteins from the cytosolic face of the ER [109]. It also protects functional and structural proteins from compression-induced oxidative stress, which is crucial for maintaining cytoskeletal integrity in cardiac muscle [41, 110, 111]. Protection by CryAB overexpression is connected with maintenance of appropriate mitochondrial protein levels, inhibition of aberrant mitochondrial permeability transition pore activation, and mitochondrial membrane potential (Δ_Ψ_) [112]. Moreover, CryAB brings protection against apoptosis through inhibiting caspase-3 activation, segregation of the antiapoptotic protein Bcl-2, and prevention of Bcl-2 translocation into the mitochondria [113]. In addition, CryAB is also inducible in response to other forms of stress such as inflammation and heat [114]. Mutations in CryAB [115], specifically the dominant *R120G* mutations in the CryAB gene (*CryAB^R120G^*), lead to myopathies via reductive stress, which is responsible for cellular hypertrophy in cardiomyocytes derived from induced pluripotent stem cells [116, 117]. *CryAB^R120G^*-induced cardiomyopathy has been established to occur along with reductive stress-induced GSH/GSSG imbalance (Figure 5) [11]. It is reported that during aging and during the progression of cardiomyopathy, both CryAB and its phosphorylation are elevated [118, 119]. The molecular tweezer CLR01 protects against *CryAB^R120G^*-induced cytotoxicity, hampers *CryAB^R120G^*-induced protein aggregation, and alleviates proteotoxicity in cardiomyocytes [120]. Besides CLR01 treatment, other recent studies state that activation of transcriptional factor EB (TFEB) by intermittent fasting also can be utilized as a treatment of *CryAB^R120G^*-induced cardiomyopathy [121]. TFEB expression and activity are dramatically vigorous during the whole course of desmin-related cardiomyopathy development. TFEB activities increase in the compensatory stage of cardiac proteinopathy and become impaired in the congestive heart failure stage. Therefore, it is important to test the effect of TFEB stimulation at any stages of cardiac proteinopathy for potential therapeutic purpose [122].

### 6.3. Nrf2-Induced Cardiomyopathy

Nrf2 is a master regulator of many cytoprotective genes [123]. Nrf2 in the heart is manipulated through a transcriptional mechanism, and its activation brings about cardioprotective effects in diverse disease models. Therefore, the Nrf2 signaling pathway is a potential target for cardiomyopathy therapy [124]. The antioxidant program regulated by Nrf2 can promote GSH synthesis and decrease intracellular ROS [125] and is thus protective against oxidative stress. Pathogenic disorders that enhance continuous stimulation of the Nrf2 response can cause reductive stress that leads to disease development [41]. Previous studies demonstrated that Nrf2-antioxidant response element signaling enhanced reductive stress in the human mutant protein aggregation cardiomyopathy (MPAC) [61]. In the MPAC-transgenic mouse model, Nrf2 scarcity was identified, which impedes ER stress and reductive stress-induced hypertrophic cardiomyopathy [126]. It should also be noted that continual activation of Nrf2 may contribute to a remarkable reduction of protein oxidation in correlation with chronic reductive stress [127]. Indeed, chronic reductive stress can exacerbate mutant protein aggregation and result in pathological cardiac remodeling, which indeed has been identified in Nrf2-transgenic mice with a constitutive activation at 10-12 weeks of age [128]. Moreover, in protein aggregation cardiomyopathy and reductive stress, the Nrf2-Kelch-like ECH-associated protein (Nrf2-Keap1) pathway is the essential transcriptional restrainer of antioxidants, proteotoxicity and isoproterenol toxicity in the heart [61, 129, 130]. Deletion of either Nrf2 or Keap1 may lead to ROS overproduction indicating the mutual control of Nrf2-Keap1 [131]. In addition, a recent study revealed that the Nrf2-Keap1 pathway is also closely related to protection against the toxicity by lead- (Pb-) induced lipid peroxidation [132]. Interestingly, another study also shows that Nrf2 level alone is capable of serving as the master regulator of the antioxidant response element without regulating the activity of Keap1, which leads to the hyperreducing power of the glutathione system [133].

## 7. Summary

In this review, we have summarized studies published mainly between 2014 and 2019, which accounts for more than four-fifth of all the citations. Further investigations should focus on identifying more reductive stress indicators as the risk assessment and prevention of developing heart damage as well as exploring more targets for cardiomyopathy treatment. Although there are still some ambiguous statements, such as the paradox between mitochondrial morphological abnormalities and mitochondrial dysfunction, we firmly believe that studying mitochondria is the critical step to reveal more unknowns. Therefore, it is expected that future investigation of underlying mechanisms of reductive stress-induced mitochondrial dysfunction will provide novel insights into therapeutic approaches for ameliorating reductive stress-induced cardiomyopathy.
